# Mammals of Australia's Tropical Savannas: A Conceptual Model of Assemblage Structure and Regulatory Factors in the Kimberley Region

**DOI:** 10.1371/journal.pone.0092341

**Published:** 2014-03-26

**Authors:** Ian J. Radford, Christopher R. Dickman, Antony N. Start, Carol Palmer, Karin Carnes, Corrin Everitt, Richard Fairman, Gordon Graham, Thalie Partridge, Allan Thomson

**Affiliations:** 1 Division of Science and Conservation, Department of Parks and Wildlife, Kununurra, Western Australia, Australia; 2 School of Biological Sciences, University of Sydney, Sydney, New South Wales, Australia; 3 Division of Science and Conservation, Department of Parks and Wildlife, Woodvale, Western Australia, Australia; 4 Department of Land Resource Management, Northern Territory Government, Palmerston, Northern Territory, Australia; 5 Conservation Commission of Western Australia, Crawley, Western Australia, Australia; 6 Department of Biological Sciences, Macquarie University, Sydney, New South Wales, Australia; University of New South Wales, Australia

## Abstract

We construct a state-and-transition model for mammals in tropical savannas in northern Australia to synthesize ecological knowledge and understand mammalian declines. We aimed to validate the existence of alternative mammal assemblage states similar to those in arid Australian grasslands, and to speculate on transition triggers. Based on the arid grassland model, we hypothesized that assemblages are partitioned across rainfall gradients and between substrates. We also predicted that assemblages typical of arid regions in boom periods would be prevalent in savannas with higher and more regular rainfall. Data from eight mammal surveys from the Kimberley region, Western Australia (1994 to 2011) were collated. Survey sites were partitioned across rainfall zones and habitats. Data allowed us to identify three assemblage states: State 0:- low numbers of mammals, State II:- dominated by omnivorous rodents and State III:- dominated by rodents and larger marsupials. Unlike arid grasslands, assemblage dominance by insectivorous dasyurids (State I) did not occur in savannas. Mammal assemblages were partitioned across rainfall zones and between substrates as predicted, but—unlike arid regions—were not related strongly to yearly rainfall. Mammal assemblage composition showed high regional stability, probably related to high annual rainfall and predictable wet season resource pulses. As a consequence, we speculate that perpetually booming assemblages in savannas allow top-down control of the ecosystem, with suppression of introduced cats by the dingo, the region's top predator. Under conditions of low or erratic productivity, imposed increasingly by intense fire regimes and introduced herbivore grazing, dingoes may not limit impacts of cats on native mammals. These interacting factors may explain contemporary declines of savanna mammals as well as historical declines in arid Australia. The cat-ecosystem productivity hypothesis raised here differs from the already-articulated cat-habitat structure hypothesis for mammal declines, and we suggest approaches for explicit testing of transition triggers for competing hypotheses.

## Introduction

The processes that drive fluctuations in population size and community composition have long been a source of fascination for ecologists, and gaining a general understanding of their identity and effects remains an enduring goal. Although some emphasis has been placed on the influence of intrinsic factors, such as social interactions, in driving species' dynamics [1], recent research has focused increasingly on the relative roles of bottom-up effects, such as resource pulses, and top-down regulatory effects, such as those induced by predators and pathogens, in shaping population and community dynamics [2], [3], [4]. The interaction of bottom-up and top-down processes is of particular interest due to the relevance of their outcomes to ‘real-world’ problems such as predicting outbreaks of pests or identifying management options for species of conservation concern [5], [6], [7].

In many systems, the interplay between bottom-up and top-down forces can lead to the existence of alternative states that are characterized by different dominant species or species-groups [8]. Alternative states may be reversible, as they are at different times in many boom-bust or pulse-reserve systems [9], or fixed, as they are in environments that have been biotically ‘homogenized’ by invasive species [10]. In situations where there is a risk of ecosystems moving toward states that are undesirable (e.g. irreversible ecosystem functional loss, species extinction), it is particularly important to identify both the drivers and thresholds beyond which recovery is not possible [11]. The identification process can be complicated if thresholds are non-linear [12], if state changes depend on the rates rather than magnitudes at which drivers have effect [13], or if transitions between states are patchy, non-synchronous and dependent on local conditions [14]. However, considerable progress has been made recently by the application of resilience-based approaches, such as state-and-transition models, that explicitly recognize the potential for ecosystems to exist as multiple alternative states [15], [8], [16].

State-and-transition models were initially developed to assist in managing non-equilibrial rangeland systems [17], but they have since been used to describe alternative states and the transitions between them in a wide range of taxa in arid, wetland and temperate forest ecosystems [18], [19], [8], [20], [21]. Rumpff et al. [22] recently suggested that state-and-transition models could be simplified and made more flexible if implemented as Bayesian networks, while several authors have extended the utility of basic descriptive models by using simulation approaches [23], [24]. Bestelmeyer et al. [25] suggested that state-and-transition models could be further improved by recognizing that factors driving state transitions may vary with spatial scale. Although these models have sometimes been criticized for being difficult to test [26], they are increasingly recognized as important tools for guiding land management [25], [21].

Here, we apply state-and-transition modelling to synthesize knowledge about native mammal assemblages in the Kimberley region of northern Australia and explore factors that might drive them. This region is of considerable conservation concern: populations of many mammal species are declining across the northern Australian savannas, conceivably towards irreversible regional or total extinctions. Worldwide, about a quarter of all mammal species are threatened with extinction; about 38% of land mammals suffer from habitat loss associated with diversion of natural resources to benefit humans, and 16% are at risk from hunting or harvesting [27]. Although diversion of natural resources is modest in northern Australia, many species in a ‘critical weight range’ (CWR: 35 g–5500 g, [28]) are undergoing steep declines in abundance and distribution. In the absence of gross anthropogenic disruption of northern Australian savannas, it has been difficult to attribute the cause(s) of these declines to any particular threatening process [29], [30].

Although mammal declines elsewhere in Australia have received considerable attention [31], a major limitation to understanding them in northern Australia has been a limited knowledge of the region's ecological systems [32], [33], [34]. Available information shows that some mammal species in the northern savannas are subject to boom-bust cycles [35], [36] similar to those typically described for desert rodents [37], [38]. Populations of other species may be influenced more by seasonal changes in the availability and quality of food [39]. However, while the dynamics of several species of savanna mammals have been described [39], [40], [41], [42], [35], [43], [44], the factors influencing those dynamics remain poorly understood. Even where observations clearly demonstrate the effects of particular processes on mammal abundance, for instance fire regimes or changes in cattle grazing intensity [33], [45], [46], insight into the mechanisms that underlie species' responses is scant (for example, whether the effects of fire relate to nutrition, predation or incineration; [47], [32], [34], [48]). As there are no long-term studies of population dynamics of northern Australian mammals, but many snap-shot surveys, synthesizing information from them offers one avenue by which state-and-transition models can be constructed.

We use a recently described model from central Australia [12] as a guide to the construction of a conceptual state-and-transition model for mammal assemblages in the tropical savannas of the Kimberley. In the arid grassland model, mammal assemblages occur in four different states, each defined by the composition and relative abundance of its constituent species. State 0 has very few mammals, State I is dominated by insectivorous (dasyurid) marsupials, State II by omnivorous rodents and State III by irruptive rodents and carnivorous marsupials. The key transition event that shifts states to higher levels (i.e. greater abundances and numbers of species) is flooding rain that produces ephemeral pulses in primary productivity, whereas processes driving the system to lower states include predation by introduced predators, drought, over-grazing by introduced herbivores, and wildfire. This model contains several ecological attributes found in the Kimberley's tropical savannas, including rainfall-driven productivity, fire and predation by native predators [49], [50], [12]. It also contains some similarities in externally-derived threatening processes such as predation by exotic predators, grazing by exotic herbivores and altered fire regimes [30]. Moreover, there is some coincidence in mammal species (e.g. *Sminthopsis macroura*, *Pseudomys desertor, Rattus villosissimus, Mus musculus*) and functional groups (particularly insectivorous dasyurids and omnivorous rodents).

There are two key differences between central Australian arid grassland assemblages and those of the Kimberley's tropical savannas: (1) The former are now largely devoid of medium-sized mammals (e.g. peramelids) whereas all medium-sized mammals persist in the tropical savannas, albeit in most cases, with greatly reduced ranges [51], [52], and (2) the Kimberley's savannas are punctuated by extensive rocky areas that could be expected to provide relatively stable and buffered conditions for mammals [53], [54].

It is important that state-and-transition models be testable [17], especially with respect to (1) demonstrating the existence of alternative states and (2) demonstrating the influence of triggers and processes that drive transitions between states [12]. In this study we address the first point by presenting data on mammal assemblages collected in the Kimberley region by surveys conducted between 1994 and 2011. We address the second point by predicting the existence of distinctive mammal assemblages, or states, characterized by differences in:

1. annual rainfall,
2. rocky versus non-rocky savannas, and
3. the abundance and representation of particular species and taxonomic or functional groups.

Because of the importance of antecedent rainfall in driving patterns of mammalian abundance and composition through time, we predicted also that inter-annual differences in rainfall would lead to shifts between states. Rainfall, and thus productivity, is predictable in the Kimberley but not in central Australia, where erratic rainfall drives ephemeral pulses of productivity. In consequence, we predicted further that Kimberley mammal assemblages would be more stable and exist in higher states than those in central Australia. We use our results to generate hypotheses about the key factors that prompt shifts between assemblage states and hence further our understanding of the threats to the Kimberley mammal faunas. These hypotheses also can be extended to, and tested in, other tropical savanna areas.

## Methods

### Study area

**Figure 1:**
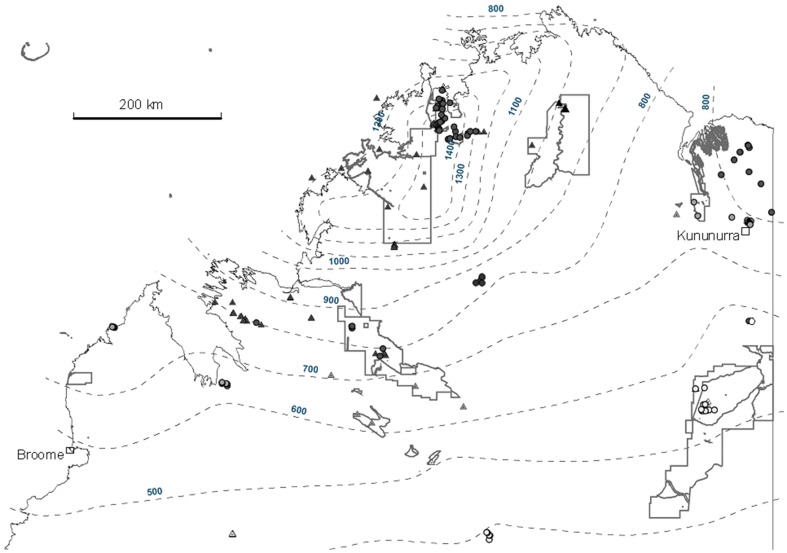
Map showing distribution of Kimberley survey sites with rainfall isohyets (grey dashed lines). Triangles and circles represent surveys in rocky and non-rocky habitats, respectively. Black, grey and pale grey represent high, medium and low rainfall sites respectively. Grey lines are conservation reserve boundaries.

The Kimberley region is in the far north west of Australia and represents the Western Australian component of Torresian (tropical savanna) habitats. It has a tropical monsoonal climate with rain falling mostly during the summer from November to April. Annual rainfall ranges from 1400 mm on the far north coast to <400 mm along the semi-arid southern boundary (Fig. 1). Although there is year to year variation in total wet season rain, rainfall is relatively predictable in its timing. Vegetation is predominantly savanna woodland with eucalypt trees dominating the canopy and tropical C_4_ grasses dominating the ground layer [55]. Geologically, the Kimberley consists of subdued but rugged sandstone, volcanic and limestone ranges that emerge from plains of alluvial, aeolian sand, and pindan sand substrates [56]. The Kimberley contains the last remaining mainland Australian region (North Kimberley Bioregion) to have experienced no known extinctions of critical weight range mammals since European colonization [57], [31], [51], [52].

### Survey stratification

To classify mammal assemblage states, we collated data from 229 survey sites in savanna across all the major Kimberley bioregions [58]. Mammal states in this context are defined as compositionally stable assemblages of co-existing mammals defined by the most common or dominant species or functional groups [12]. To gain information on the role of production pulses (rainfall) in triggering transitions, we stratified sites into three rainfall zones: a semi-arid low rainfall zone (<600 mm annual rainfall), a medium rainfall zone (600–800 mm) and a high rainfall zone (>800 mm) (Fig. 1). We further stratified sites into those set up on rocky or non-rocky substrates [59], [60], [61]. In this study we did not attempt to undertake tests of additional transition triggers including fire, domestic livestock or predator interactions.

### Survey designs

All survey data presented here consist of one-off site surveys. Where repeat site surveys occurred, we maintained temporal independence by choosing just one survey at random to represent that site. As different rainfall regions and savanna habitats provide the potential conditions needed to drive different states and transitions, we considered this space-for-time approach to be appropriate.

This study makes use of data from eight separate mammal fauna surveys with differing methodologies and trap effort per site (Table 1). Despite these differences, we consider it unlikely that they would have affected our ability to describe mammal assemblages. All but one survey used Elliott traps (large: 15×15.5×46 cm and medium sized: 9×10×33 cm) baited with peanut butter and oats, sometimes with additional ingredients (Table 1). Cage traps (c. 30×50×60 cm, baited with peanut butter and oats) (1∶5 cages to Elliotts) were also used in some surveys, mostly in the medium and high rainfall zones (Table 1) where larger CWR mammals are still extant [31], [51]. A few pitfall traps (1∶10 pits to Elliotts) suitable for passive capture of small rodents and dasyurids (<35 g) were included in some surveys across all rainfall zones (Table 1). The mean trap-effort per site varied among surveys (from 84 to 2032 trap nights; Table 1), leading to some variation in our ability to detect rare species at individual sites. For this reason our data do not necessarily represent comprehensive species lists for sites, but rather indexes of relative abundance for more common species. Survey design varied from transects [51], [52] to quadrats [62] to trapping grids [48] (Table 1). These designs potentially yield different estimates of abundance. However, as our objective was to describe broad regional-scale assemblage patterns in terms of dominant mammal species and functional groups, we do not consider these differences in survey design/methodology to be important.

**Table 1:** Data custodians, dates, publication details, Kimberley zones trapped and methods used to obtain survey data included in this study.

Custodian^1^	Study	Date(s)	Publication	Zone^2^	Methods^3^
ADF/DPAW	Anon.	1995	Unpublished	HR	El, P
					tr(183)*10
DPAW	Carnes	2007–2009	Unpublished	HR, MR, LR	El., C, P
					w(128)*10
DPAW	Everitt	2009–2010	Unpublished	HR, MR	El, C, P
					tr(2032)*10
DPAW	Graham	1994 a, b	Unpublished	MR	P
					tr(1360)*2
NHT/DPAW	Palmer	2001–2002	Palmer [95]	HR, MR, LR	El, C, P
					w(84)*78
Macquarie	Partridge &	2004–2005	Partridge [96]	LR	El, P
Uni/DPAW	Thomson				tr(912)*18
DPAW	Radford &	2007–2011	Radford [48]	HR, MR	El, C, P
	Fairman		& unpb.		g,w(114)*79
DPAW	Start &	2003–2004	Start *et al*. [51]	HR, MR, LR	El, C
	Palmer		[52]		tr(594)*22

1 ADF, Australian Defence Force; DPAW, Department of Parks and Wildlife (and predecessors); NHT, Natural Heritage Trust; ^2^HR, high rainfall zone, MR, medium rainfall zone, LR, low rainfall zone; ^3^El, Elliott traps; C, cage traps; P, pitfall traps; tr, transect-based trapping [51]; w, Woinarski plot-based trapping [62]; g, grid-based trapping [48]. unpb, unpublished. Numbers in parentheses in the right hand column refer to the mean number of trap nights per survey site and the number following is the total number of survey sites for which data are presented.

### Statistical analyses

Analyses were performed on individual species and also on pre-defined functional groups (small dasyurid insectivores (<35 g), terrestrial omnivorous rodents (Muridae), arboreal rodents, large marsupials (>150 g) including dasyurid predators, bandicoots (peramelids), possums (pseudocheirids and phalangerids), and small macropods (macropodids <2000 g)). We compared species and taxonomic/functional groups in terms of their abundance, measured here as percentage trap success (no. animals/trap nights ×100) for each survey. We tested for potential under-estimation of small dasyurids among surveys by separately analyzing trap records from pitfall traps based on the rationale that pitfall traps are the most effective method for estimating the abundance of small dasyurids in arid grassland surveys [63].

The hypothesis that species and mammal groups differ in abundance among rainfall zones and between rocky and non-rocky savannas was tested using Kruskal-Wallis tests. This non-parametric test was chosen due to inequality of sample sizes and non-normality of the survey data, which had many zero values. The effect of fluctuations in annual rainfall within rainfall zones/savanna types (used in Kruskal-Wallis tests, above) was tested using regressions of trap success over previous wet season rainfall. Differences among mammal assemblages across rainfall zones and savanna habitats were explored using Bray Curtis ordinations, with the Sorensen dissimilarity distance used to calculate separation between surveys. A non-parametric multi-response permutation procedure (MRPP) was used to test for differences among predefined rainfall zone and savanna habitat (rocky and non-rocky) groups. We used PC-ORD 4 for ordinations and MINITAB 14 for all statistical analyses.

## Results

### Kimberley mammal assemblages

After removal of data from sites sampled more than once, we recorded a total of 5138 mammals from 71,016 trap nights (mean trap success 7.24%) (Table 2). The largest mammal group comprised omnivorous rodents, with a trap success of 5.08%. The most common rodents in order of abundance were *Zyzomys argurus, Pseudomys nanus, Rattus tunneyi* and *P. delicatulus*. The next most prominent group comprised large dasyurid predators (*Dasyurus hallucatus* and *Phascogale tapoatafa*), with 0.95% trap success. Small insectivorous dasyurids were relatively rare and yielded a total trap success of only 0.09%. Other groups represented in the surveys included bandicoots (0.58%) and arboreal rodents (0.43%). The low trap success rate for macropods and possums (<0.2%) probably reflects ineffective sampling rather than low numbers as these animals are not often trapped even where locally numerous.

**Table 2:** Trap success (%) as an index of abundance for Kimberley mammal species caught during surveys conducted from 1994 to 2011.

	Frequency among surveys	High rainfall rocky	Medium rainfall rocky	Low rainfall rocky	High rainfall non-rocky	Medium rainfall non-rocky	Low rainfall non-rocky	*H*	*P*
Site no.	Total = 229	74	8	21	68	37	21		
Omnivorous rodents									
* Hydromys chrysogaster*	1	0	0	0	0.001	0	0	2.37	0.796
* Leggadina lakedownensis*	6	0.019	0.036	0	0.007	0	0	18.10	0.003**
* Melomys burtoni*	9	0.162	0	0	0.344	0	0	6.07	0.299
* Mus musculus* *	8	0	0.016	0.050	0.195	0	0	12.41	0.030*
* Pseudomys delicatulus*	67	0.437	0.091	0.066	0.415	1.631	1.124	24.45	<0.001***
* Pseudomys nanus*	71	0.290	0.295	0.145	0.890	0.877	7.51	33.67	<0.001***
* Pseudomys desertor*	7	0	0	0.730	0	0	0	71.18	<0.001***
* Pseudomys johnsoni*	4	0.019	0	0.031	0	0	0	21.40	0.001**
* Rattus rattus* *	1	0	0	0	0.038	0	0	5.19	0.393
* Rattus tunneyi*	54	0.968	0.357	0.006	0.937	0.429	0.267	15.49	0.008**
* Rattus villosissimus*	4	0	0.075	0	0.004	0	0	28.81	<0.001***
* Zyzomys argurus*	75	5.326	0.810	0.375	0.384	0.253	0	79.10	<0.001***
* Zyzomys woodwardi*	21	0.888	0	0	0	0	0	48.07	<0.001***
subtotal		8.110	1.681	1.404	3.150	3.228	8.90	27.86	<0.001***
Arboreal rodents									
* Conilurus penicillatus*	4	0.002	0	0	0.330	0	0	4.46	0.486
* Mesembryomys macrurus*	16	0.948	0	0	0.091	0	0	16.58	0.005**
subtotal		0.950	0	0	0.421	0	0	16.17	0.006**
Dasyurid predators									
* Dasyurus hallucatus*	59	2.573	0	0	0.283	0	0	58.36	<0.001***
* Phascogale tapoatafa*	3	0.113	0	0	0	0	0	6.34	0.275
subtotal		2.686	0	0	0.283	0	0	99.62	<0.001***
Peramelids (bandicoots)									
* Isoodon auratus*	22	0.696	0	0	0	0	0	50.59	<0.001***
* Isoodon macrourus*	37	0.648	0.018	0	0.502	0	0	27.56	<0.001***
subtotal		1.344	0.018	0	0.502	0	0	53.42	<0.001***
Insectivorous dasyurids									
* Planigale ingrami*	4	0.019	0.009	0	0	0.039	0	10.08	0.073
* Planigale maculata*	11	0.019	0	0.080	0.009	0	0	29.94	<0.001***
* Pseudantechinus ningbing*	5	0	0.017	0.066	0.003	0	0	9.02	0.101
* Sminthopsis virginiae*	6	0	0	0	0.115	0	0	14.52	0.013*
* Sminthopsis macroura*	13	0	0.009	0.112	0	0.002	0.084	64.40	<0.001***
subtotal		0.038	0.035	0.262	0.126	0.035	0.084	33.00	<0.001***
Pseudocheirids & phalangerids (possums)									
* Petropseudes dahli*	6	0.019	0.106	0	0	0	0	41.93	<0.001***
* Trichosurus vulpecula*	1	0	0.018	0	0	0	0	27.62	<0.001***
* Wyulda squamicaudata*	5	0.060	0	0	0	0	0	10.66	0.059
subtotal		0.085	0.124	0	0	0	0	31.20	<0.001***
Small macropods									
* Petrogale burbidgei*	4	0.030	0	0	0	0	0	8.49	0.131
* Petrogale concinna*	7	0.086	0.018	0	0	0	0	13.28	0.021*
subtotal		0.116	0.018	0	0	0	0	18.65	0.002**
TOTAL MAMMALS	188	13.350.	2.102	1.732	4.487	3.291	8.980	49.30	<0.001***
SPECIES RICHNESS		3.500	4.625	1.381	1.971	1.324	1.714	55.11	<0.001***

*Introduced species. Statistics are for the Kruskal-Wallis (*H*) test and *P* values <0.05 indicate significant differences among rainfall zones and savanna habitats (rocky and non-rocky).

Although not recorded by trapping, dingoes *Canis lupus dingo* and feral cats *Felis catus* were abundant. Dingoes were frequently observed and recorded in the high and medium rainfall zones during surveys (e.g. Radford 2012, unpublished data). Similarly, cats are present throughout the Kimberley [64], were often detected in high and medium rainfall zone surveys (Radford 2012 unpublished data), and are abundant in areas such as Purnululu National Park in the low rainfall zone.

### Do rainfall zone and savanna type influence mammal abundance?

Species and group abundances, assessed using trap success, varied strongly among rainfall zones and rocky and non-rocky savannas. Small CWR mammals (<150 g, including omnivorous rodents and insectivorous dasyurids) showed variable responses to rainfall and habitat. Some rodent species (e.g. *P. delicatulus, P. nanus*) were more numerous in low rainfall zones and non-rocky savannas, while others (e.g. *Z. argurus, Z. woodwardi*) were most abundant in high rainfall rocky savannas (Table 2). Some rodents were restricted with regard to savanna type and rainfall zone. For instance, *P. desertor* was found only in low rainfall rocky savannas, while *Leggadina lakedownensis* occurred in high and medium rainfall savannas (Table 2).

**Figure 2:**
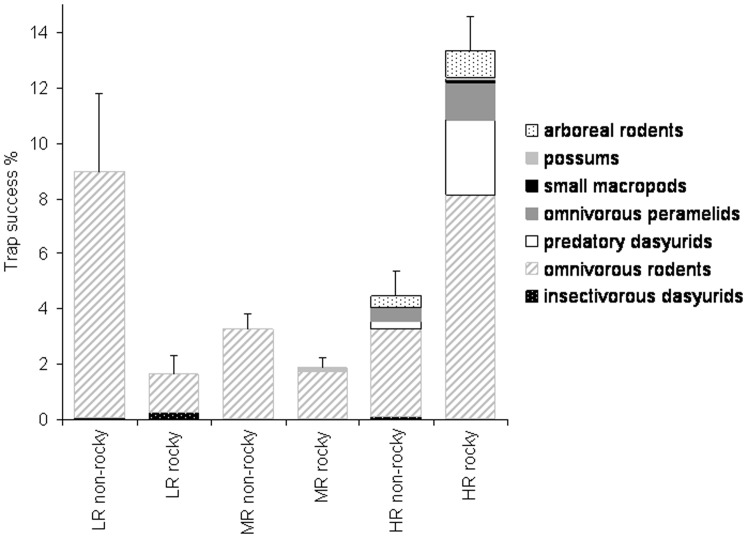
Trap success (mean + SE) of the major mammal functional groups (see key) recorded in different habitats in surveys across the Kimberley, northern Australia. Habitat types are defined by rainfall zone (H  =  High (>800 mm), M  =  Medium (600–800 mm) and L  =  Low (<600 mm)) and savanna habitat structure (rocky or non-rocky).

Like the rodents, small insectivorous dasyurids varied in their response to rainfall and savanna habitat. *Sminthopsis virginiae* was found only in high rainfall non-rocky savannas, while *S. macroura* was most abundant where rainfall was low (Table 2). Unlike rodents and small dasyurids, larger mammals (>150 g, arboreal rodents, dasyurid predators, bandicoots, small macropods, possums) were consistently more numerous in high rainfall, rocky savannas (Table 2, Fig. 2). Large dasyurid predators (*D. hallucatus*), the bandicoot *Isoodon auratus* and arboreal rodents occurred only in surveys in the high rainfall zone (Table 2, Fig. 2). Possums, small macropods and the bandicoot *I. macrourus* occurred only in rocky savannas in high and medium rainfall zones (Table 2). Total mammal abundance was highest in high rainfall rocky savannas, although mean rodent abundance (and variability measured by standard error) was higher in low rainfall non-rocky savannas (Table 2, Fig. 2). Mammal species richness was highest in high and medium rainfall rocky savannas.

### Do differences in yearly rainfall influence mammal abundance?

**Figure 3:**
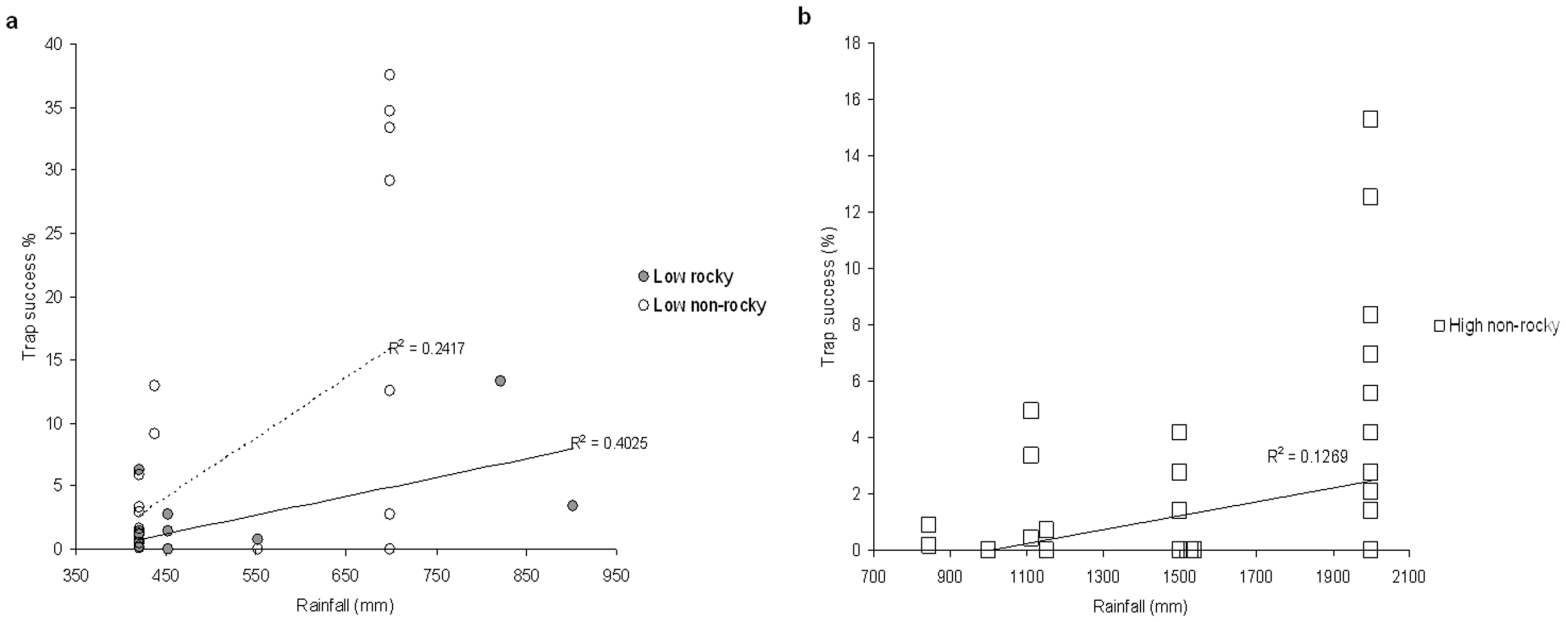
Trap success of a) all mammals in low rainfall rocky and non-rocky savannas and b) critical weight range mammals (non-rodent) in high rainfall woodlands (non-rocky) in the Kimberley, northern Australia. Rainfall is plotted as the amount of rain received in the wet season prior to sampling.

Annual variation in rainfall had some influence on mammal abundance in addition to the effects of rainfall zone and type of savanna habitat. Total mammal trap success was positively influenced by previous wet season rainfall in the low rainfall zone (Table 3, Fig. 3a) in both rocky and non-rocky savannas. Similarly, the trap success of combined large-bodied mammals (>150 g, dasyurid predators, bandicoots, arboreal rodents, possums, macropods) was related positively to variation in annual rainfall in non-rocky savannas in the high rainfall zone (Table 3, Fig. 3b). No other regressions for other mammal groups by habitat or rainfall zone were significant (Table 3).

**Table 3:** Regressions of total trap success for all mammals and for mammals >150 g of the Kimberley with rainfall preceding the year of survey for different rainfall zones and savanna habitats (rocky and non-rocky).

	Total trap success (%)			Trap success, mammals >150 g (%)		
	D.F.	F	*P*	D.F.	F	*P*
High rainfall (>800 mm)						
rocky	1, 72	1.38	0.244	1, 72	2.95	0.090
non-rocky	1, 66	2.74	0.102	1, 66	9.59	0.003**
Medium rainfall (600–800 mm)						
rocky	1, 6	1.41	0.280	1, 6	0.26	0.631
non-rocky	1, 35	0.04	0.851	-	-	-
Low rainfall (<600 mm)						
rocky	1, 19	12.80	0.002**	-	-	-
non-rocky	1, 19	6.06	0.024*	-	-	-

D.F., degrees of freedom; F, F statistic for Analysis of Variance; *P*, *P* significance value for Analysis of Variance.

### Do rainfall zone and savanna type influence mammal assemblage composition?

**Figure 4:**
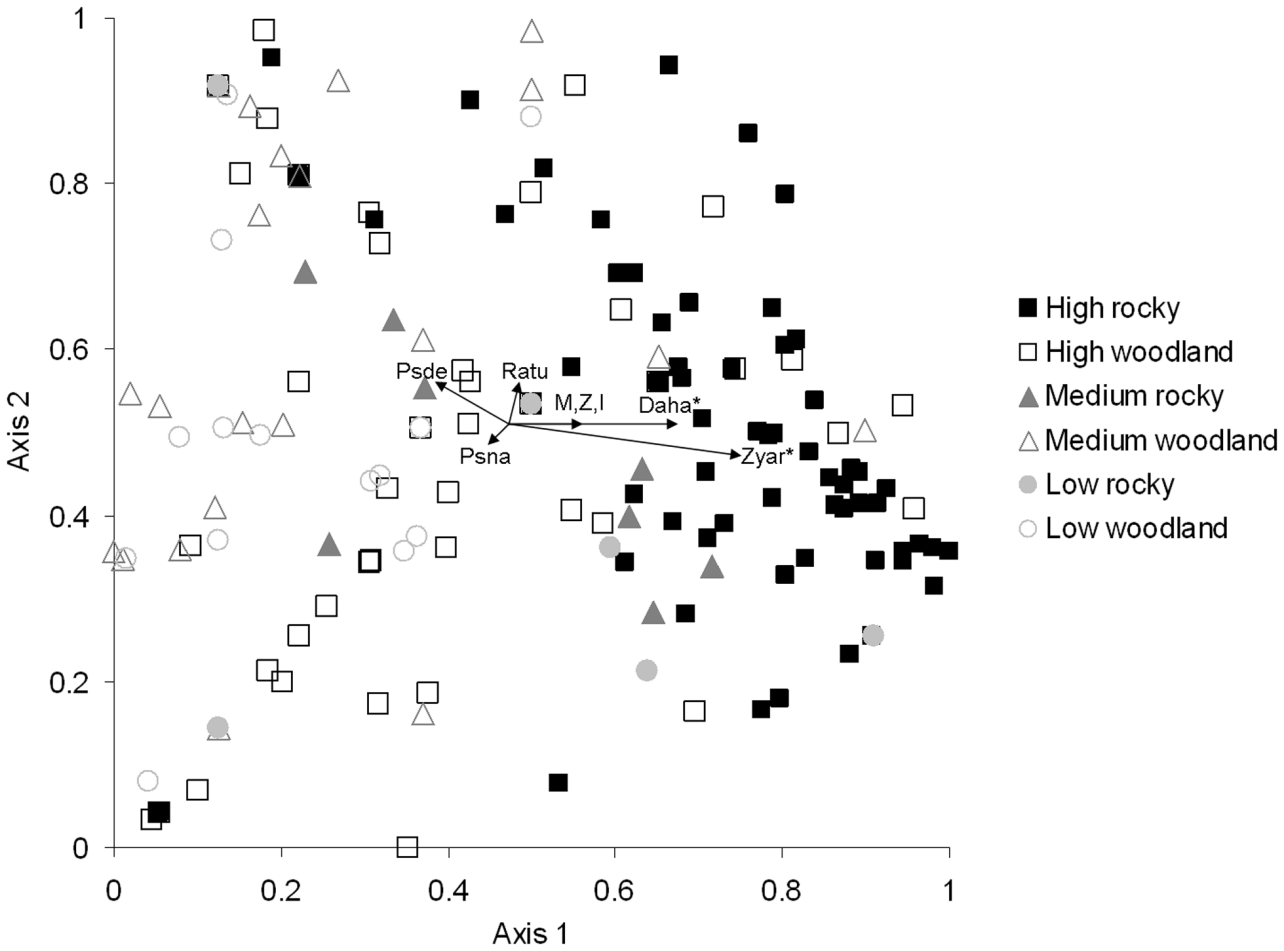
Bray-Curtis ordination of mammal survey data from across rainfall zones and major habitat types in the Kimberley, northern Australia, from 1994 to 2011. In the legend, “High”, “Medium” and “Low” refer to rainfall zones (high >800 mm, medium 600–800 mm, low <600 mm), and rocky and non-rocky refer to the two major savanna habitat types among survey sites. Vector lines refer to all Pearson correlations of *r*>0.05, with significant correlations (*P*<0.05) denoted by ‘*’. Letter codes shown on the ordination refer to species names that correlate with ordination axes: ‘Daha’  =  *Dasyurus hallucatus*; ‘Zyar’  =  *Zyzomys argurus*; ‘Psde’  =  *Pseudomys delicatulus*; ‘Psna’  =  *Pseudomys nanus*; ‘Ratu’  =  *Rattus tunneyi*; and ‘M,Z,I’  =  *Mesembriomys macrurus, Zyzomys woodwardi* and *Isoodon auratus*, respectively.

Mammal assemblages differed significantly among rainfall zone/savanna habitat type combinations (MRPP, *T* = −27.01; *A* = 0.098; *P*<0.001; Fig. 4). High rainfall rocky assemblages clustered positively along axis 1 of the Bray Curtis ordination (Fig. 4). *Zyzomys argurus, D. hallucatus, M. macrurus, I. auratus* and *Z. woodwardi* were correlated positively with this ordination axis, indicating they are more abundant in these high rainfall rocky savannas (Fig. 4). Rocky savanna assemblages in the low and medium rainfall zones did not segregate strongly from rocky high rainfall savannas on the basis of assemblage structure (Fig. 4). Non-rocky savannas in all rainfall zones were correlated negatively along axis 1, with high rainfall sites correlated negatively, and medium and low rainfall sites correlated positively, with axis 2 (Fig. 4). *Pseudomys nanus* was more abundant in high rainfall non-rocky savannas while *P. delicatulus* was more abundant in low and medium rainfall non-rocky savannas. *Rattus tunneyi* was positively correlated with axis 2 of the ordination, and was associated with a mixture of savanna habitats (Fig. 4).

### Do trapping methods bias measurement of mammal assemblage composition?

**Figure 5:**
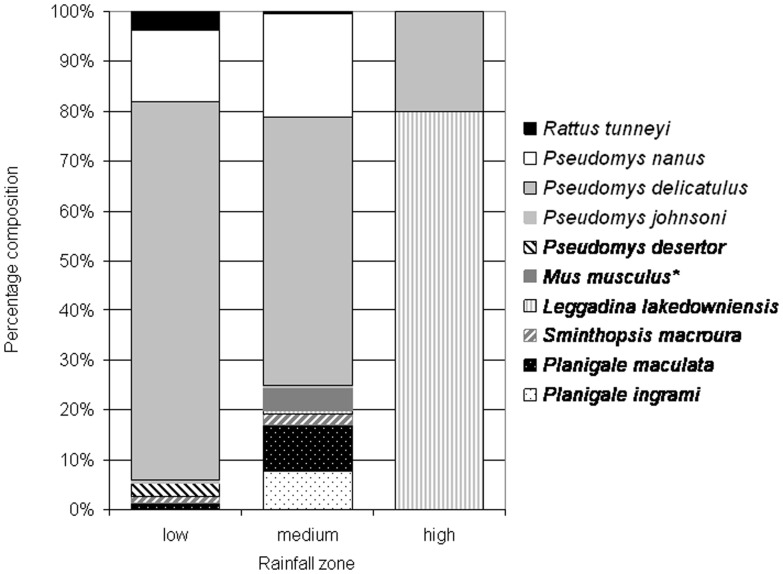
Mammal species data from pitfall traps set in the Kimberley, northern Australia, where data could be separated from other trapping information. Trapping results are shown for different percentages of total captures. Pitfall trap data were provided by the Australian Defence Force, Thomson, Partridge, Graham, Radford and Everitt.

Pitfall trap records confirm that our surveys, which were dominated by Elliott trap data, did not markedly underestimate abundances of small dasyurids. Only smaller mammals (<150 g), including rodents and small dasyurids, were captured using pitfall traps irrespective of rainfall zone (Fig. 5). Rodents made up most pitfall records (≥80%) in all surveys, while insectivorous dasyurids in all zones comprised a maximum of only 20% of captures (0% captures in the high rainfall zone, Fig. 5).

### Defining mammal states in Kimberley savannas

In individual surveys, 17% of sites had no mammals (State 0), 2% were dominated by insectivorous dasyurids (State I), 37% were dominated by omnivorous rodents (State II) and 44% had a significant component of large mammals in addition to omnivorous rodents (State III). Surveys yielding zero mammals (State 0) were unusual in high (8% of surveys) and medium (0%) rainfall rocky savannas but more common in non-rocky savannas (24%, 27% and 21% in the high, medium and low rainfall zones, respectively) and in low rainfall rocky savannas (32%). Small dasyurids (State I) dominated assemblages in 1% of high rainfall rocky savannas, and in 5% and 10% of rocky and non-rocky sites in the low rainfall zone. Rodents dominated assemblages (State II) in medium rainfall non-rocky (73%), low rainfall rocky (63%) and low rainfall non-rocky (71%) savannas. State II assemblages were also common in medium rainfall rocky (25%) and high rainfall non-rocky savannas (38%), but less so (6%) in high rainfall rocky savannas. State III assemblages with large CWR mammals (>150 g) were restricted to high rainfall or rocky savannas, with 84% and 75% of surveys yielding these assemblages in high and medium rainfall rocky savannas, and 40% in high rainfall non-rocky savannas.

## Discussion

### Validation of mammal assemblage states in tropical savannas

**Figure 6:**
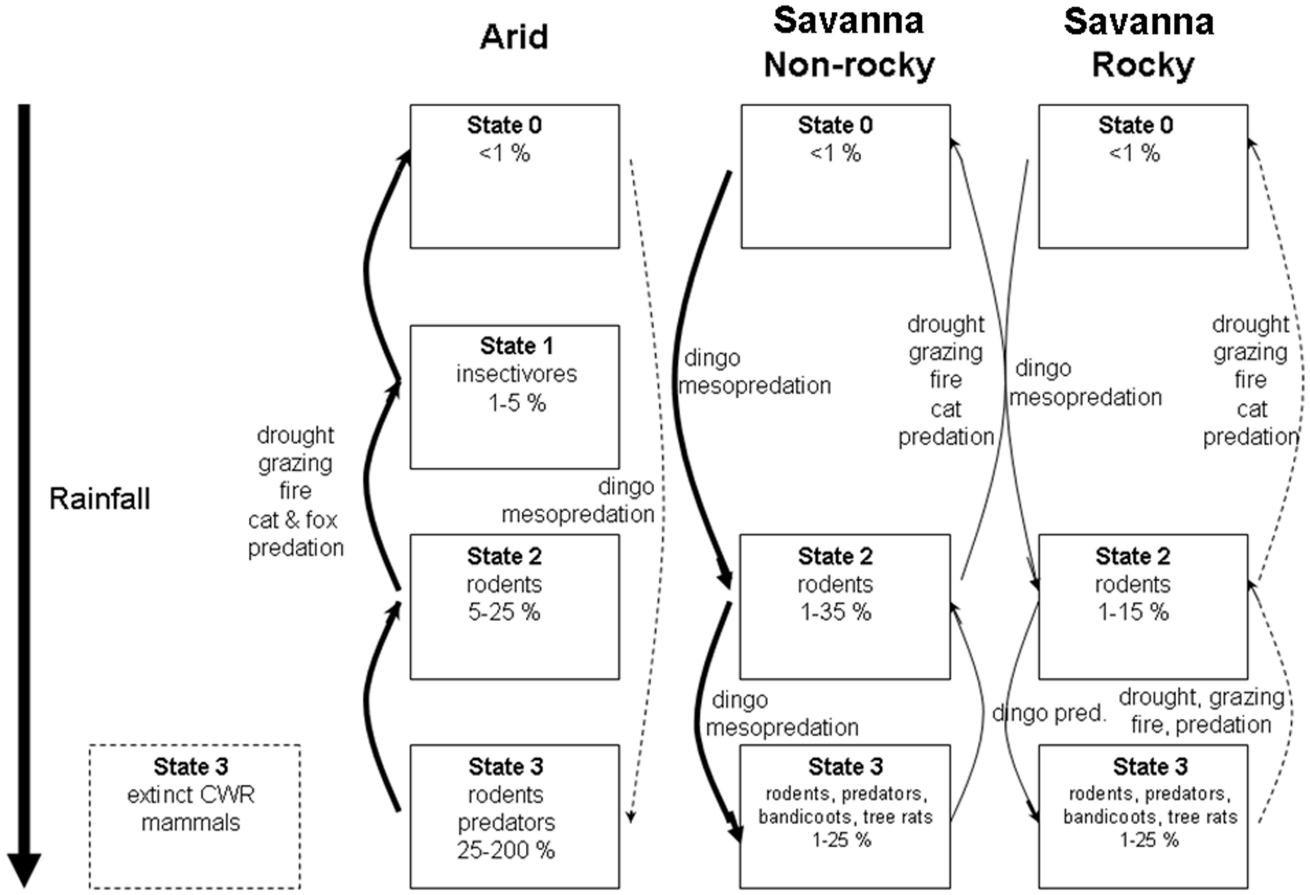
State-and-transition models for assemblages of small- and medium-sized mammals in arid and savanna habitats. States are indicated by rectangles and transitions that drive state-changes are shown by arrows. Solid arrows are known transitions, dotted arrows represent putative transitions identified in the present work. The arid model is based on Letnic and Dickman [12], the savanna models on the results of the present study. Percentage values within rectangles represent average total trap success rates for species within the respective states. State I (comprising insectivorous marsupials) is absent from the Kimberley savannas, whereas several critical weight range (CWR: 35–5500 g) mammals that were part of State 3 assemblages in arid habitats, but which are now extinct, are shown in the rectangle defined by the dashed line.

This study confirms the existence of alternative assemblage states for mammals in tropical savannas of the Kimberley region. A proposed model depicting these states is shown in Fig. 6. A State 0 assemblage, equivalent to arid zone State 0 with very low abundance of mammals (very low or zero trap success) and no dominant group, was observed at many Kimberley survey sites. This assemblage was most common in the low rainfall semi-arid zone (30% of survey sites) and less common in the high rainfall zone (<10%). State II assemblages, with omnivorous rodent species as the dominant group [12], were observed commonly across the Kimberley, particularly within the low and medium rainfall zones. State II savanna assemblages on rocky substrates were dominated by *Z. argurus* and *P. desertor* and on non-rocky substrates (sand plains and open non-rocky savannas) by *P. nanus, P. delicatulus* and *R. tunneyi*. These species differ from their arid zone counterparts where State II assemblages are dominated by *Notomys alexis* and *Pseudomys hermannsburgensis* (Letnic and Dickman 2010). Finally, surveys in the high rainfall zone conformed to State III arid zone assemblages, with rodents and marsupial groups including predatory dasyurids dominating. State III assemblages were dominated by rodents (*Z. argurus, Z. woodwardi* or *P. nanus, R. tunneyi* and *Melomys burtoni*) and the large dasyurid predator *Dasyurus hallucatus* on rocky substrates. State III assemblages also included bandicoots (*Isoodon macrourus* and *I. auratus*), arboreal rodents (*Mesembriomys macrurus* and *Conilurus penicillatus*) and infrequently trapped possums (e.g. *Wyulda squamicaudata*) and small macropods (*Petrogale* spp.). The latter groups, comprising CWR mammals, have become extinct since European settlement in central Australian grasslands [49], [57], Fig. 6).

A key difference between the arid zone assemblages described by Letnic and Dickman [12] and those described here is that a functional State I assemblage consisting mainly of insectivorous dasyurids was missing. Small insectivorous dasyurids including *Sminthopsis macroura, S. virginiae, Planigale ingrami, P. maculata* and *Pseudantechinus ningbing* occurred throughout Kimberley. However, they never occurred at high abundance, nor were they ever more common than other mammal groups. In contrast, arid assemblages are often dominated by small dasyurids (i.e. State I) [12].

The assemblages described here correspond closely with previous reports on mammals in the Kimberley savannas [65], [59], [61], [51], [31], [45], [46], [52], [48] and in savannas of the Northern Territory [60], [29], [66], [67], [33], [62]. Dasyurid predators (*D. hallucatus*, *P. tapoafata*), bandicoots and arboreal rodents have generally been restricted to high rainfall savannas in the Kimberley and Top End regions since the 1980s [39], [60], [61], [67], [31], [51], [52], [48]. Differences between rocky and non-rocky savanna assemblages have been reported [60], [61], with small dasyurids generally being quite scarce [39], [59], [60], [29], [66], [62], [48]. Recent surveys in medium and low rainfall zones of the Kimberley [65], [45], [46], [52] also show the loss of State III assemblages (large non-rodents) from these regions. Similar patterns of disappearance of non-rodent CWR fauna have been documented for Northern Territory savannas [30]. Offshore island assemblages in high and medium rainfall areas of the Kimberley [68] and the Northern Territory [69] are variously dominated by omnivorous rodents (State II) or large marsupials (State III), similar to our findings reported here.

### Transition and regulation processes in the savanna biome

Key transition triggers for changes in mammal assemblage state in central Australian grasslands are rainfall events that drive ephemeral pulses of primary production, and wildfires, drought, grazing by introduced herbivores and predation by introduced predators (cats and foxes) [12]. Similar to the arid grassland transitions, assemblages of Kimberley savanna mammals also differed across regional rainfall gradients. As described above, State III assemblages were confined mostly to the North Kimberley high rainfall zone, whereas State II and State 0 assemblages predominated in the medium and low rainfall zones.

Despite the predictability of these patterns, assemblage states were not fixed; for example, State II assemblages sometimes occurred in the high rainfall zone and State III assemblages in the medium rainfall zone. Several other studies have also documented changes in assemblage states at particular sites over time [33], [45], [48]. For example, McKenzie [31] recorded historic changes among central Kimberley assemblages in the medium and low rainfall zones from State III to State II prior to the 1980s. Surveys in the 1980s in the low rainfall zone at Purnululu recorded the historical presence of State III species (e.g. *D. hallucatus*) [70] even though there is no evidence of their presence in recent surveys. Many high rainfall savannas in the Northern Territory have recently transitioned from predominantly State III assemblages to State II or State 0 assemblages as many larger marsupials and rodents disappeared from surveys [62], [30]. Conversely, changes from State II to State III occurred at some sites in the North Kimberley between 2007 and 2012 with increases in abundance of large marsupials and rodents (Radford unpublished report 2011, Myers unpublished data 2012). Despite these changes, we found little evidence for frequent or rapid transitions between assemblage states in the Kimberley due to annual changes in rainfall, although abundances of some mammals, especially in the low rainfall zone, did increase following wet years. We also found no evidence of the order-of-magnitude changes in mammal abundance that characterize arid zone assemblages (Fig. 6).

High regional stability among assemblages of Kimberley savanna mammals in response to inter-annual differences in rainfall suggests that transitions there are governed differently from those in central Australian grasslands. Because savanna productivity is generally relatively high due to higher rainfall than in more arid regions, factors that effect downward state transitions may assume more importance in driving assemblage changes. Such factors operating in the savannas may also explain the relatively low abundance of mammals in most Kimberley assemblages relative to those in the arid grasslands during periods of population irruption [49], [50]. We consider these factors further below.

### Predation

Predation by introduced cats and foxes drives mammal assemblages towards lower states in arid regions [57], [7], [71], particularly in areas where predator populations are boosted by the presence of abundant introduced prey species such as rabbits [50]. In the Kimberley savannas, by contrast, foxes do not occur and native top-predators such as dingoes, raptors, pythons and goannas may suppress or interfere with cat populations, thereby reducing their impacts on native species and allowing mammal groups no longer present elsewhere to persist [72], [71], [73]. The dominant role of top-predators in regulating ecosystems worldwide is becoming increasingly evident [74], [75], [76], [77], [78], [71], particularly in productive regions [5], [79].

Of the predators in the Kimberley savannas, dingoes are most likely to have a top-predator role and to influence mammal assemblage states. Ecological theory and many empirical observations show that where resource pulses are regular, as in the Kimberley relative to the arid zone, predators often maintain higher and more stable populations and can then exert strong regulatory effects on prey [5], [6], [79]. Under such conditions dingoes could affect cat densities or hunting behaviour [80], [72], [73]. Dingoes may also benefit native mammal assemblages by suppressing the dominant competitors of CWR mammals or by suppressing herbivores such as large macropods [74], thereby increasing vegetation cover or complexity and hence shelter [6], [81], [71]. Strong top-down effects exerted by dingoes in consistently productive savanna habitats would also provide a tenable explanation for the inverse relationship between historical mammal extinctions and ecosystem productivity in Australia [31]. In arid ecosystems, dingoes may still suppress smaller introduced predators during periods of resource stasis, but temporarily lose their regulatory effects after flood rains when populations of prey increase so rapidly that they escape top-down suppression [7], [71].

If dingoes act as ‘biodiversity regulators’ [71] in Kimberley savannas, increases in primary productivity (e.g. from irrigation, reduced fire or cattle impacts) could be expected to increase dingo density, reduce cat predation and facilitate transitions to higher mammal assemblage states [82]. Conversely, declines in primary productivity should lead to decreases in dingo numbers, increased impacts from cat predation and cause transition to lower assemblage states. Experimental increase and stabilization of dingo populations (e.g. by provision of supplementary food or removal of poison baiting) also should prompt transitions to higher mammal assemblage states (if non-rodent mammals are present), while reductions in dingo numbers should drive transitions to lower states. While the role of dingoes in influencing cat activity is being investigated [71], [72], [73], experiments will likely be needed in future to reliably quantify links between dingoes, cat predation and savanna mammal assemblages in northern Australia. Meanwhile, however, by providing a mechanistic explanation for how mammal assemblages are structured in the tropical savannas, the predictive tests of transition triggers that we suggest should provide useful tools for conservation managers seeking to slow mammal declines in the region.

### Fire and introduced herbivores

As in the conceptual model for arid Australian assemblages, fire regimes and introduced herbivores have major influences on assemblages of savanna mammals [33], [45], [46]. In arid ecosystems, fire and cattle both reduce the productivity available to mammals; they also reduce ground cover, which may increase exposure to introduced predators such as cats [57], [12], [48]. In the Kimberley, severe fires temporarily deplete ground layer vegetation biomass and cover [48], reduce soil nutrient levels [83], lower the availability of resources such as grass seeds and invertebrate prey [84], [85], [86], affect woody plant structure and biomass [87], [88] and savanna net primary productivity [89], [90]. Changes in fire regimes resulting in more extensive, frequent and high intensity fires as reported for northern Australia in recent decades [91] have likely led to reduced savanna net primary productivity. Increasing populations of large introduced herbivores such as domestic cattle, feral camels, buffalo, donkeys and horses can also potentially reduce primary productivity. Sustained grazing by large herbivores reduces herbaceous biomass and results in selection for less palatable, lower productivity herbaceous species [92]. Large herbivores can also exacerbate fire impacts on primary productivity through preferential feeding in recently burnt habitats [93], [94]. Increased episodic removal of savanna productivity through increased severity of fire regimes and increased grazing impacts through increasing populations of introduced herbivores may prompt transitions to lower mammal assemblage states if these assemblages are linked to net primary productivity.

### Implications

Arid zone mammals typically occur as State 0 or State I assemblages for long periods until shifted up by large but infrequent flooding rains [12], whereas mammals of the Kimberley savannas—sustained by predictable wet season rainfall—can be characterized mostly as State II or State III assemblages. Recent losses of mammals from some parts of northern Australia that have shown shifts in assemblages to lower states have not been caused by the failure of wet season rainfall [29], [30]. However, our findings here suggest a two-step process that may effect such assemblage changes. Firstly, recent intensification of fires and the effects of grazing by introduced herbivores in the Kimberley would reduce environmental productivity and place downward pressure on native mammal assemblages. Secondly, a decrease in the magnitude, spatial and temporal predictability of environmental productivity would weaken top-down control of the system by the dingo and facilitate increased impact on native mammals by the introduced feral cat. The marked historical declines of mammals in Australia's arid regions took place under conditions of low and erratic productivity and during periods when top-down suppression of smaller introduced predators would have been weakened by the active control of dingoes [50], [71]. We propose that similar interactions may now be taking place in Northern Territory and Kimberley savannas and acting in concert to affect mammal abundances and assemblage composition.

The cat-ecosystem productivity hypothesis posited here, and depicted in Fig. 6, invokes similar factors to an explanation for mammal declines proposed by Johnson [57], but differs in its mechanism. Johnson's [57] proposal identifies habitat simplification by fire and introduced herbivores as the cause of the historically high impacts of cats on mammals in arid regions and more recently in savannas. Although dingoes may still suppress cat activity under this hypothesis, their indirect positive effects on small native species are relatively small compared to the benefits that cats experience from hunting these prey in structurally simplified habitats. Under the cat-ecosystem productivity hypothesis, by contrast, cats do not benefit so much from the exposure of their prey in simplified habitats, but rather from the negative effects of reduced ecosystem productivity on the dingo. If dingoes have suppressive effects on cats and cat suppression in turn is needed to halt the continuing decline of savanna mammals, a simple test of our hypothesis is possible: in the presence of experimentally stabilized dingo populations, native mammal assemblages should remain intact or recover to higher states. Although dingoes are reviled in many areas owing to their attacks on livestock [71], critical experiments initially could be small-scale and carried out on conservation estate.
